# Functional Characterization of *Acinetobacter baumannii* Lacking the RNA Chaperone Hfq

**DOI:** 10.3389/fmicb.2017.02068

**Published:** 2017-10-27

**Authors:** Han-Yueh Kuo, Hsuan-Hao Chao, Po-Cheng Liao, Long Hsu, Kai-Chih Chang, Chi-Hua Tung, Chang-Hua Chen, Ming-Li Liou

**Affiliations:** ^1^Department of Medicine, National Taiwan University Hospital Hsin-Chu Branch, Hsinchu, Taiwan; ^2^Department of Internal Medicine, College of Medicine, National Taiwan University, Taipei, Taiwan; ^3^Department of Electrophysics, National Chiao Tung University, Hsinchu, Taiwan; ^4^Department of Medical Laboratory Science and Biotechnology, Yuanpei University, Hsinchu, Taiwan; ^5^Department of Laboratory Medicine and Biotechnology, Tzu Chi University, Hualien, Taiwan; ^6^Department of Bioinformatics, Chung Hua University, Hsinchu, Taiwan; ^7^Division of Infectious Disease, Department of Internal Medicine, Changhua Christian Hospital, Changhua, Taiwan; ^8^Center of Infection Prevention and Control, Changhua Christian Hospital, Changhua, Taiwan

**Keywords:** *Acinetobacter baumannii*, Hfq, adhesion, invasion, outer membrane vesicles

## Abstract

The RNA chaperone Hfq is involved in the riboregulation of diverse genes via small RNAs. Recent studies have demonstrated that Hfq contributes to the stress response and the virulence of several pathogens, and the roles of Hfq vary among bacterial species. Here, we attempted to elucidate the role of Hfq in *Acinetobacter baumannii* ATCC 17978. In the absence of *hfq*, *A. baumannii* exhibited retarded cell growth and was highly sensitive to environmental stress, including osmotic and oxidative pressure, pH, and temperature. Compared to the wild-type, the Hfq mutant had reduced outer membrane vesicles secretion and fimbriae production as visualized by atomic force microscopy. The absence of *hfq* reduced biofilm formation, airway epithelial cell adhesion and invasion, and survival in macrophage. Further, the *hfq* mutant induced significantly higher IL-8 levels in airway epithelial cells, which would promote bacterial clearance by the host. In addition to results similar to those reported for other bacteria, our findings demonstrate that Hfq is required in the regulation of the iron-acquisition system via downregulating the *bauA* and *basD* genes, the stress-related outer membrane proteins *carO*, *A1S_0820*, *ompA*, and *nlpE*, and the stress-related cytosolic proteins *uspA* and *groEL*. Our data indicate that Hfq plays a critical role in environmental adaptation and virulence in *A. baumannii* by modulating stress responses, surface architectures, and virulence factors. This study is the first to illustrate the functional role of Hfq in *A. baumannii.*

## Introduction

*Acinetobacter baumannii* has emerged recently as a major cause of healthcare-associated infections worldwide (Maragakis and Perl, 2008). These organisms have been implicated in a diverse range of infections in hospitalized patients, especially patients with prolonged stays in intensive care units (Consales et al., 2011). The widespread capability of this organism may depend on the expression of virulence factors that enable bacterial infection and the expression of antimicrobial-resistance determinants (Giamarellou et al., 2008; Gordon and Wareham, 2010). Several studies have investigated the mechanisms of pathogenicity and antimicrobial resistance contributing to the astonishing success of this pathogen. The remarkable resistance phenotype may be facilitated by the ability of *A. baumannii* strains, particularly those isolated from catheter-related urinary or bloodstream infections, to form biofilms that adhere to and persist on abiotic surfaces (Lee et al., 2008). The ability of *A. baumannii* to adhere to a number of epithelial cell lines has recently been investigated (Lee et al., 2006; Giannouli et al., 2013). Following adhesion, *A. baumannii* is able to invade and promote apoptosis of eukaryotic cells via OmpA (Omp36), which is trafficked to both the mitochondria and the nucleus and induces eukaryotic cell death pathways (Choi et al., 2008). The ability of *A. baumannii* to obtain and utilize resources such as iron via the siderophore acinetobactin is an another factor in survival both in the host and in the environment (Zimbler et al., 2009). Although a number of novel genes in *A. baumannii* with significant roles in pathogenesis have been discovered (Smith et al., 2007; Vallenet et al., 2008), the intrinsic regulatory mechanisms involved in environmental adaptation and disease progression remain unclear.

Post-transcriptional regulation of gene expression based on small non-coding RNA molecules (sRNAs), which is usually involved in the response to environmental stress to maintain cell homeostasis, has become a focus of interest in bacteria (Storz et al., 2011). sRNAs are usually 50–300 nucleotides long and modulate mRNA translation and/or stability by complementary base-pairing (Waters and Storz, 2009). One key player for many sRNA–mRNA interactions is the RNA chaperone Hfq (Vogel and Luisi, 2011). The Hfq protein, which belongs to the eukaryotic family of Sm proteins, was first discovered in *Escherichia coli* as a host bacterial factor required for RNA synthesis of bacteriophage Qβ (Franze de Fernandez et al., 1968). Hfq oligomerizes into a hexameric ring structure (Sauter et al., 2003) that facilitates sRNA–mRNA interactions and contributes to RNA regulation by interacting with the RNA turnover enzymes RNase E, polynucleotide phosphorylase, and poly(A) polymerase (Vogel and Luisi, 2011). Hfq is now regarded as a transcriptional regulator involved in stress responses, iron homeostasis, and outer membrane biogenesis (Chao and Vogel, 2010).

The influence of Hfq on physiology and virulence has been studied in a number of Gram-positive and Gram-negative bacteria (Sittka et al., 2007; Simonsen et al., 2011; Wang et al., 2014). Using *hfq* null mutants and *hfq* complementation strains, it has become clear that Hfq generally modulates motility and promotes resistance to cellular stresses such as oxidative stress or low pH (Chao and Vogel, 2010), but *hfq* mutation results in diverse phenotypic changes in several bacterial species (Bohn et al., 2007; Sittka et al., 2007). Cellular genes involved in protection from nutrient deprivation, oxidative, and alcoholic stresses have been demonstrated in *A. baumannii* (Fiester and Actis, 2013). For example, several genes involved in the acinetobactin-mediated iron acquisition system, such a*s bas*, *bau*, and *bar*, play roles in the ability of *A. baumannii* to cause infections in hosts that impose iron-limiting stress (Gaddy et al., 2012). In addition, genes involved in protection from oxidative stressors such as the universal stress protein A (*uspA*) (Elhosseiny et al., 2015), *nlpE* (Dorel et al., 2006), A1S_0820 (Buist et al., 2008), *carO* (Mussi et al., 2007), *groEL* (Soares et al., 2010), and *ompA* (Soares et al., 2010) have been identified. Although Hfq plays critical roles in the resistance to cellular stresses, the correlation between Hfq and these stress-related molecules in *A. baumannii* remains unclear.

The role of Hfq in *A. baumannii* is not known. The aim of this study was to clarify the function of the Hfq homolog of *A. baumannii* in virulence and stress responses. We constructed a *hfq*^-^ mutant of the *A. baumannii* 17978 type strain by gene replacement and a *hfq*-complemented strain. We demonstrated that loss of *hfq* affects a number of virulence-associated phenotypes, including bacterial morphology, biofilm formation, resistance to stress response, cell adhesion, and invasion ability, in *A. baumannii* ATCC17978. In addition, we demonstrated that loss of *hfq* affects transcription of genes involved in stress response. This is the first report of the functional roles of *A. baumannii* Hfq.

## Materials and Methods

### Bacterial Strains, Plasmids, and Primers

The bacterial strains and plasmids used in this study are listed in **Table 1**. The primers used in this study are listed in Supplementary Table S1. Bacteria were routinely cultured at 37°C in Mueller-Hinton (MH) medium at 37°C with constant shaking unless otherwise indicated.

**Table 1:** Bacterial strains and plasmids used in this study.

Strain or plasmid	Genotype and/or characteristics	Reference or source
***A. baumannii* strains**		
ATCC 17978	Wild-type strain	ATCC^a^
Δhfq	Δ*hfq*	This study
Hfqc	Δ*hfq* harboring pHFQ	This study
*E. coli*		
DH5α	Used for recombinant DNA methods	Invitrogen
**Plasmid**		
pWH1266_km	Amp^r^ Tc^r^ Km^r^; expression vector^b^	Zimbler et al., 2009
pGEM-T	High-copy-number cloning vector; Amp^r^	GMBiolab, Taichung, Taiwan
pHFQ	pWH1266_km harboring the 17978 *hfq* allele	This study


*^a^American Type Culture Collection (Manassas, VA, United States). ^b^Amp^*r*^, ampicillin resistance; Tc^*r*^, tetracycline resistance; Km^*r*^, kanamycin resistance.*

### Replacement of the *A. baumannii hfq* Gene

The whole genome sequence of *A. baumannii* ATCC17978 was obtained from GenBank (Smith et al., 2007) and adopted to manipulate the *A. baumannii* ATCC 17978 genome. The hfq::Km mutants were constructed by a previously described method (Aranda et al., 2010). Briefly, polymerase chain reaction (PCR) was used to amplify regions of the sequences upstream and downstream of the *hfq* gene using the primer pairs (Supplementary Table S1) Up_Hfq_F and Hfq_kan_R to amplify the 311-bp region upstream of the *hfq* gene and Kan_Hfq_F and Dw_Hfq_R to amplify the 220-bp region downstream of the *hfq* gene. The primers Hfq_kan_R and Kan_Hfq_F have approximately 22 nucleotides of overlapping sequence, resulting in fusion of the hfq upstream and downstream sequences with the kanamycin resistance gene after fusion PCR. The 311 and 220-bp DNA fragments containing the overlapping sequences from the *km* gene were fused together by PCR with a complementary *km* PCR fragment, resulting in the replacement of *hfq* with *km*. The resulting PCR product was gel purified using a gel extraction kit (Favorgen, PIF, Taiwan). The fused PCR fragment was electroporated into wild-type *A. baumannii* ATCC 17978. The Δhfq strains were identified by screening transformants on LB agar plates containing kanamycin (50 μg/μl). The deletion mutant was confirmed using conventional PCR for diagnostic size shifts by gel electrophoresis and was further confirmed by real-time reverse transcription PCR (RT-PCR).

### Complementation Study

The *hfq* parental allele was PCR amplified using the primers hfq_Expr_XhoI_F and hfq_Expr_XbaI_R and ligated into the *A. baumannii*–*E. coli* shuttle vector pWH1266_km (Lin et al., 2014). The recombinant DNA products were verified by sequencing. The resulting plasmid pHFQ was used to transform the Δhfq strain by electroporation with selection by tetracycline. The expression of the *hfq* gene in the Δhfq mutant and its complementation clone Δhfqc was verified using real-time RT-PCR by the method previously described (Chang et al., 2014). The relative expression level of *hfq* was normalized using that of 16s rRNA.

### Atomic Force Microscopy (AFM)

Overnight bacterial cultures were diluted 1:100 and subcultured for 3 h. The bacteria were recovered by centrifugation at 3,000*g* for 10 min, washed with deionized water, and suspended in deionized water at an OD_600_ of 0.07. Droplets of each sample were deposited on glass slides for 10 min. Then, the droplets were removed, and the slides were dried for 20 min to immobilize the cells. The morphology of the bacteria was analyzed by atomic force microscopy (AFM; Dimension Icon, Bruker, Billerica, MA, United States) using an SNL-W-D triangular cantilever probe (Bruker) with a nominal 0.06 N/m spring constant and 2-nm tip curvature radius. Measurements were obtained in peak force tapping mode (or Quantitative NanoMechanics mode, QNM mode). The images obtained were analyzed using NanoScope Analysis 1.4 software (Bruker). The AFM scanning sizes were 3 μm × 3 μm with scan rates of 1 Hz. The spatial resolution of image is 5.86 nm/pixel. For each measurement, height sensor and peak force error images were recorded simultaneously. The height sensor image specifies fimbria morphology, while the peak force error image emphasizes edges of bacterial structures.

### Isolation of *A. baumannii* OMVs

Outer membrane vesicles (OMVs) are isolated using the method of Rosen et al. (1995). Protein concentrations were determined and then equal volume of OMV content in each strain was analyzed by 10% SDS–PAGE.

### Growth Curves

*A. baumannii* strain ATCC17978 (wild-type) and the Δhfq and Δhfqc strains were cultured overnight in MH medium at 37°C, and the overnight culture was subcultured at 1:100 in 100 ml of MH medium. The cultures were incubated with shaking at 200 rpm in 250-ml Scott flasks at 37°C for 22 h. Every hour, the optical density was measured at 600 nm (OD_600_).

### Stress Tolerance Assays

For the stress tolerance test, overnight cultures were diluted to an OD_600_ of 0.01 with MH broth. For temperature effects, cells were cultured at 20 and 50°C, respectively. For high osmolarity and oxidative stress effects, NaCl and H_2_O_2_ were added to the bacterial cell suspension at concentrations of 3% and 20 mM, respectively. In addition, ethanol and Triton X-100 were added to the bacterial suspension at concentrations of 3 and 1%, respectively. For acid and alkaline stress conditions, cells were cultured at pH 5.5 and pH 8.5, respectively. Bacterial growth with or without external stress was monitored by measuring the OD_600_. After treatment with H_2_O_2_ for 30 min, the cells were diluted and plated on LB plates to determine the number of colony forming units (CFUs). The results represent the mean of at least three separate experiments.

### Evaluation of Stress-Related Gene Expression by Real-Time RT-PCR

To study the effect of the *hfq* mutation on the mRNA levels of *basD, bauA, uspA*, *nlpE*, A1S_0820, *carO*, *groEL*, and *ompA*, overnight cultures of the wild-type, Δhfq, and Δhfqc strains were washed, and total RNA was isolated for real-time RT-PCR.

### Biofilm Assays and Evaluation of Fimbria Expression by Real-Time PCR (qPCR)

Biofilm formation on polystyrene tubes was assessed by crystal violet staining of cells cultured in LB broth as previously described. The expression of the *csuA/B* gene (A1S_2218) and fim-like adhesion gene (A1S_1507) in the Δhfq mutant and its complementation clone Δhfqc was verified using real-time RT-PCR by the method previously described (Chang et al., 2014). The relative expression levels of A1S_2218 and A1S_1507 were normalized by that of 16s rRNA.

### Intra-Macrophage Survival Assay

The assay was performed as described with some modifications (Wang et al., 2014). Briefly, mouse macrophage cells (RAW264.7) were cultured to 1 × 10^6^ cells/well in 24-well plates in RPMI 1640 medium. The overnight culture of *A. baumannii* was applied to each well at an MOI of 10. Bacteria were co-incubated with macrophages for 120 min at 37°C. After infection, the cells were washed and incubated for 1 h with streptomycin (250 μg/ml) containing medium. The CFU obtained from the lysate of the wild-type-infected macrophages was set as 100%, and other data were relative to this value.

### Cell Adhesion and Invasion Assays

Cell adhesion and invasion assays were performed as described with some modification (Wang et al., 2014). A549 (Human lung carcinoma cell line, epithelial cell; BCRC 60074) and NCI-H292 (human mucoepidermoid pulmonary carcinoma cell line, endothelial cell; BCRC 60372) were purchased from Food Industry Research and Development Institute, Hsinchu, Taiwan. Briefly, overnight bacterial cultures were diluted 100-fold and grown for 2 h. A549 cells and NCI-H292 cells were infected at 37°C for 2 h with bacterial suspensions at a multiplicity of infection (MOI) of 10. For the adhesion assay, infected monolayers were washed with PBS to remove non-adherent bacteria and lysed with 1% Triton X-100. The cell lysates were plated on LB agar plates to determine the total CFU associated with the cells. For the invasion assay, the infected cells were washed and further incubated at 37°C for 2 h in RPMI 1640 medium containing streptomycin (500 μg/ml) to kill extracellular bacteria. Cells were lysed and plated on LB agar to quantify viable invading bacteria. The adhesion ability was expressed as the percentage of adherent bacteria versus the total inoculum, and the invasion ability was expressed as the percentage of viable bacteria that survived the streptomycin treatment versus the total inoculum.

### Cytokine Evaluation by Real-Time RT-PCR

Overnight bacterial cultures were diluted 100-fold and grown for 2 h. A549 cells and H292 cells were infected at 37°C for 1 h at an MOI of 10. The infected cells were collected, and total RNA was isolated using an RNeasy minikit (Qiagen). The mRNA expression of IL-8 and IL-6 in A549 cells and H292 cells was determined by real-time RT-PCR as described (Kim et al., 2015). The relative amounts of cytokines in each infected cell line were normalized to the expression levels of the GAPDH gene.

### Statistical Analysis

The means of group differences were determined using a one-way ANOVA. A *p*-value < 0.05 was considered statistically significant. Data entry and analyses were performed using the Statistical Package for the Social Sciences software version 21.0 (SPSS Inc., Chicago, IL, United States).

## Results

### Construction of the *A. baumannii hfq* Mutant

**FIGURE 1:**
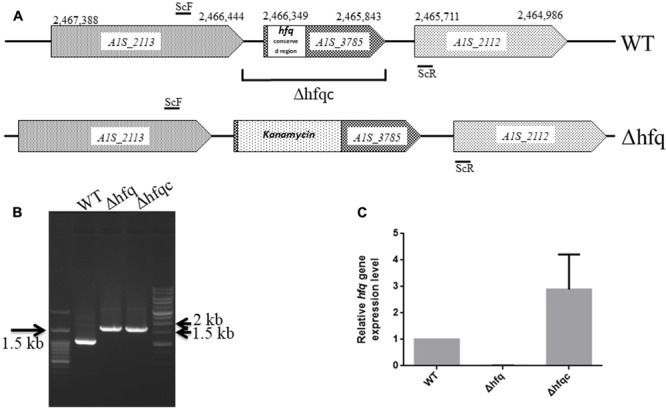
Mutational analysis of the *A. baumannii* ATCC 17978 *hfq* gene. **(A)** Schematic representation of the linear DNA constructed for the *hfq* gene replacement. The numbering denotes the gene coordinates in the *A. baumannii* genome database. In the Δhfq mutant, the conserved region (1–66 amino acid) of the Hfq ORF was replaced by the kanamycin resistance gene. The DNA fragment cloned in the complementation plasmid in Δhfqc is indicated. The primers used are listed in Supplementary Table S1. **(B)** Screening of *hfq A. baumannii* mutants generated by gene replacement. WT, wild-type control with 994 bp. Δhfq and Δhfqc (from kanamycin replacement) with 1,636 bp. **(C)** Hfq mRNA expression analyzed by real-time RT-PCR.

Several studies have demonstrated that Hfq participates in the pathogenesis of many bacteria via different mechanisms. Here, we studied the effects of an Hfq-like protein on the pathogenesis of *A. baumannii* ATCC 17978. The gene (A1S_3785) homolog of the *hfq* gene, which is predicted to encode a protein of 168 amino acids, is located at 2,465,843–2,466,349 bp in the genome of *A. baumannii* ATCC17978 (Eijkelkamp et al., 2011). This gene (A1S_3785) and its encoded protein are almost twice the size of other gammaproteobacterial Hfqs due to an elongated C-terminus (Supplementary Figure S1). The N-terminal domains of Hfq in most bacteria are highly conserved between amino acid residues 1 and 66 (Supplementary Figure S1), a region that is responsible for RNA-binding and protein–protein interactions among Sm [the core of small nuclear ribonucleoprotein particles (snRNPs)] and Sm-like proteins (Moller et al., 2002). By contrast, the C-terminal end of *A. baumannii* Hfq contains a glycine-rich domain with a repetitive pattern, which was similar to the *A. baylyi hfq* gene encoding a usual C-terminus (Schilling and Gerischer, 2009). Compared to the Hfq protein lengths in several bacteria, the largest known Hfq proteins are annotated in members of the gammaproteobacterial family *Moraxellaceae* (Supplementary Table S1). The annotated Hfq lengths are between 168 and 174 amino acids for Acinetobacter and up to 210 amino acids for *M. catarrhalis.* Although Hfq shows a strong variation in its C-terminus (Vecerek et al., 2008), this C-terminus of Hfq in *A. baylyi* was not responsible for growth and cell phenotype (Schilling and Gerischer, 2009). To examine the role of Hfq in *A. baumannii*, we generated an isogenic mutant by the replacing the conserved *hfq* sequence with the kanamycin resistance gene (Δhfq) and an Hfq-complemented strain (Δhfqc), as shown in **Figure 1A**. The loss of *hfq* was verified by PCR and sequencing (**Figure 1B**). The loss and recovery of *hfq* expression was confirmed in the Δhfq and Δhfqc strains by real-time RT-PCR (**Figure 1C**).

### Visualization of Cell Morphology

**FIGURE 2:**
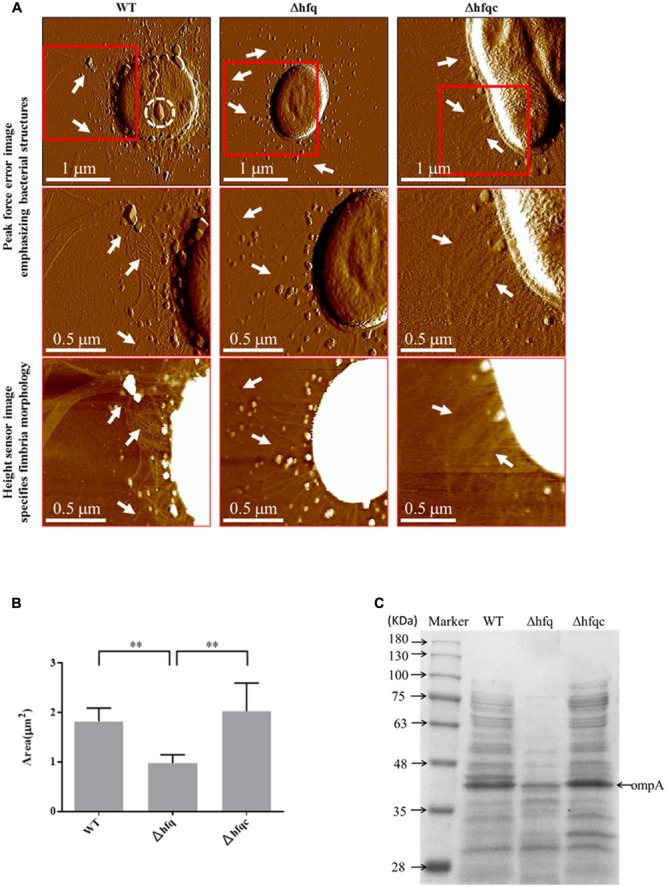
Cell morphology visualization. **(A)** Cell morphology visualization by AFM. Columns from left to right are WT, Δhfq, and Δhfqc. The first row shows the peak force error images of the three strains. The second row and third row are the peak force error images and the height sensor images of the red square region of the first row, respectively. The white arrow indicates fimbriae while the dashed white circle indicates outer membrane vesicles (OMV). Numbers of cells analyzed for WT, Δhfq, and Δhfqc are 6, 13, and 7, respectively. **(B)** Inactivation of Hfq leads to decreased cell size. ^∗∗^*p* < 0.01. **(C)** Equal volume of total protein content of *A. baumannii* OMVs was subjected to 10% SDS–PAGE.

The cell morphologies of the three *A. baumannii* strains (WT, Δhfq, and Δhfqc) were visualized from the AFM peak force error images, as shown in **Figure 2A**. Δhfq expressed very few fimbriae (white arrow), compared to WT and Δhfqc strains. **Figure 2B** shows the comparison of the cell sizes by estimating the bacterial spreading areas from the AFM height sensor images as shown in **Figure 2A**. The cell area (A) and number (*n*) are as following: WT (A: 1.82 ± 0.27 μm^2^, *n*: 5) and Δhfqc (A: 2.03 ± 0.57 μm^2^, *n*: 5) strains were both significantly larger than Δhfq (A: 0.98 ± 0.17 μm^2^, *n*: 6) (*p* < 0.01). This result reveals that a lack of *hfq* could restrain the growth of *A. baumannii.* There was no significant size difference between WT and Δhfqc. OMVs (dashed white circle) were dominant in wild-type and were greatly reduced in the Δhfq strain.

To verify that Δhfq strain reduced OMVs secretion, the quantitative assay of OMVs was performed and the result was shown in **Figure 2C**. The total protein content of OMVs was largely reduced in Δhfq strain compared to WT and Δhfqc strains. Based on the study of Jin et al. (2011), OmpA of *A. baumannii* is the most abundant protein in OMVs with a molecular mass of 38 KDa. Taken together, the levels of OmpA were reduced in Δhfq strain compared to WT and Δhfqc strain.

### Growth Effects on *hfq* Deletion Mutants

**FIGURE 3:**
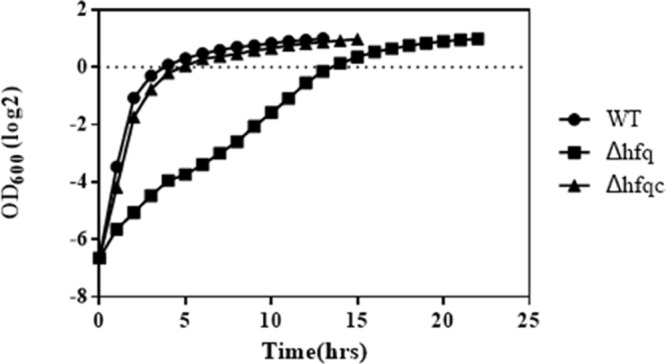
Growth of *A. baumannii* Δhfq in Mueller-Hinton medium. *A. baumannii* wild-type, Δhfq and Δhfqc cells were cultured in MH broth at 37°C. The OD600 values of each strain were measured hourly. The error bars represent the means and standard errors of the mean (SEM) of three independent replicates.

Growth defects resulting from the loss of *hfq* have been demonstrated in several bacterial species (Sonnleitner et al., 2003; Geng et al., 2009; Wang et al., 2014). To evaluate the growth effects in *hfq* deletion mutants, we monitored the growth of the wild-type, Δhfq mutant, and Δhfqc strains in MH broth. The loss of *hfq* resulted in growth retardation of 7 h (**Figure 3**). On MH agar plates, except for colony size, other colony characteristics such as form, elevation, and margin did not differ between *hfq* mutant and wild-type strain (data not shown).

### The Effect of Hfq on Stress Tolerance

**FIGURE 4:**
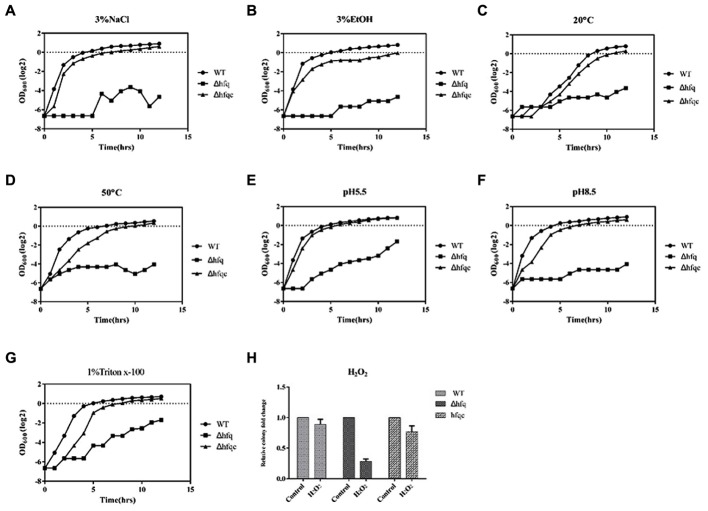
The effect of Hfq on stress tolerance of *A. baumannii.* Tolerance to **(A)** 3% NaCl; **(B)** 3% ethanol; **(C)** 20°C; **(D)** 50°C; **(E)** pH 5.5; **(F)** pH 8.5; **(G)** 1% Triton X-100; **(H)** 20 mM H_2_O_2._ Bacterial cultures in exponential phase were adjusted to 10^8^ cells/ml with or without external stress. Bacterial growth was monitored by measuring the OD_600_. The numbers of bacteria surviving H_2_O_2_ stress were measured, and the relative cell survival was obtained relative to the untreated control. All data represent the average of three independent experiments with the SD.

*Acinetobacter baumannii* has gradually become a leading nosocomial pathogen worldwide. The widespread dissemination of *A. baumannii* in hospital environments indicates that this organism might tolerate environmental stresses. To determine if Hfq contributes to external stress tolerance, the growth of the WT, Δhfq, and Δhfqc strains was assessed under various stress conditions. Hfq mutants showed defects in growth upon exposure to all stressors (**Figure 4**). The reduced tolerance of the Hfq mutant was restored to near wild-type levels by introduction of the Hfq-expressing plasmid. This result implies that Hfq contributes to the resistance to stresses such as temperature, pH, osmotic pressure, and oxidative stress in *A. baumannii*.

### The Effect of Hfq on the mRNA Expression of Stress-Related Molecules

**FIGURE 5:**
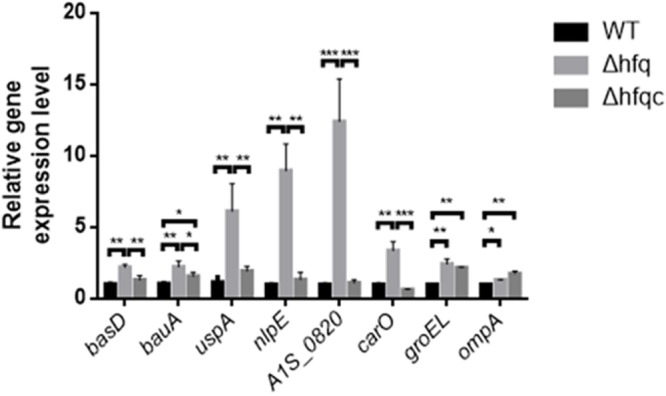
The effect of Hfq on the expression of stress-related signaling molecules. The relative mRNA expression of the *basD, bauA, uspA, nlpE*, *A1S_0820, carO, groEL*, and *ompA* genes in the WT, Δhfq, and Δhfqc strains was determined by real-time RT-PCR. All data represent the average of three independent experiments with the SD. ^∗^*p* < 0.05; ^∗∗^*p* < 0.01; ^∗∗∗^*p* < 0.001.

As Hfq contributes to external stress tolerance, the effect of Hfq on the mRNA expression of the genes involved in stress tolerance, including the acinetobactin-mediated iron acquisition system such a*s bas* and *bau*, and growth-related stresses such as *uspA*, *nlpE*, A1S_0820, *carO*, *groEL*, and *ompA*, was evaluated (**Figure 5**). Our result showed that the expression of all genes, including *basD*, *bauA*, *uspA*, *nlpE*, *A1S_0820*, *carO*, *ompA*, and *groEL*, was significantly higher in Δhfq (*p* < 0.05) than in WT and was completely or partially restored in the Δhfqc strain. These data indicate that Hfq is required to modulate the expression of stress-related genes to survive stress.

### The Effect of Hfq on Biofilm Formation and Fimbria Production, Adhesion, Invasion, and Cytokine Production in Lung Epithelial Cells

**FIGURE 6:**
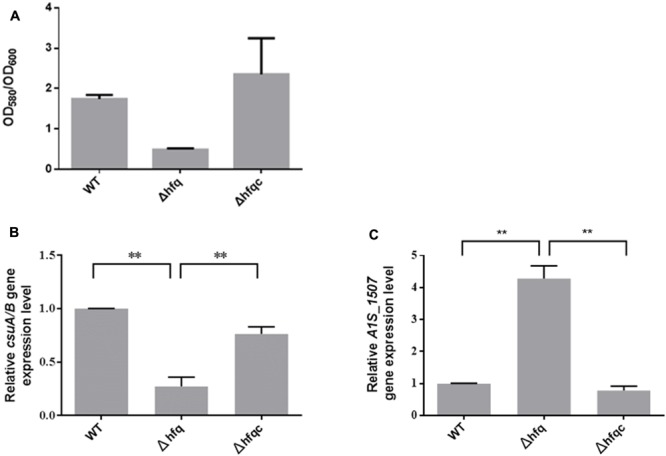
The effect of Hfq on biofilm formation and fimbria expression. **(A)** Biofilm formation in the WT, Δhfq, and Δhfqc strains. The biofilm level was determined as described in the Section “Materials and Methods.” **(B)** Three dimension features of bacterial biofilm structure visualized by scanning electron microscopy. **(C)** Relative mRNA expression of *csuA/B* and *A1S_1507* was analyzed using real-time RT-PCR. All data represent the average of three independent experiments with the SD. ^∗∗^*p* < 0.01.

*Acinetobacter baumannii* form biofilms, a phenotype that may explain its ability to survive in nosocomial environments and to cause device-related infections in compromised patients (Lee et al., 2008). As shown in **Figure 6A**, when *hfq* was deleted, biofilm formation was somewhat decreased compared with wild-type. Fimbria assembly plays an important role in biofilm initiation and maturation after initial attachment to abiotic surfaces (Gaddy and Actis, 2009). Two types of fimbria, the CsuA/B-A-B-C-D-E chaperone-usher secretion system and fim-like type 1 fimbria (A1S-1507 to A1S_1510), have been reported in *A. baumannii* (Tomaras et al., 2003; Nait Chabane et al., 2014). CsuA/BABCDE-dependent fimbriae are involved in biofilm formation, whereas the function of fim-like fimbria remains unclear. To determine if Hfq plays a role in fimbriae production, the expression levels of *csuA/B* and A1S_1507 in the wild-type and Δhfq and Δhfqc strains were examined by real-time RT-PCR as shown in **Figure 6B**. CsuA/B levels were significantly reduced (*p* < 0.01), whereas A1S_1507 levels were significantly increased (*p* < 0.01) in the Δhfq strain compared with the wild-type and Δhfqc strain, indicating that Hfq is required to modulate fimbriae production in different ways.

### Deletion of *hfq* Affects the Adhesion, Invasion, and Cytokine Production of Airway Epithelial Cells

**FIGURE 7:**
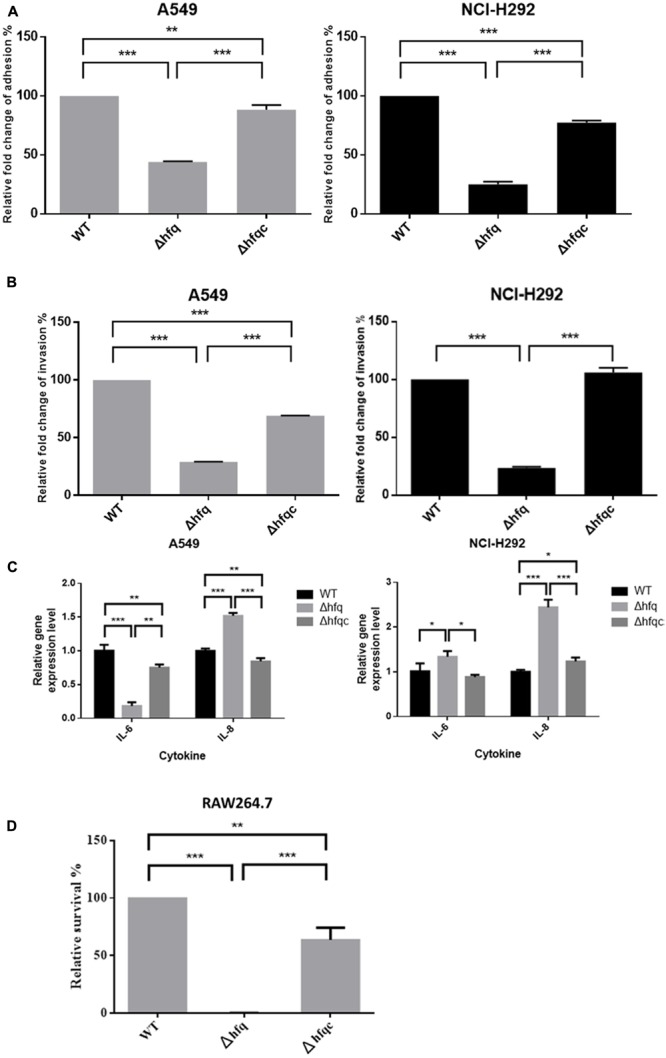
The effect of Hfq on the adhesion, invasion, cytokine secretion of airway epithelial cells and survival in macrophage. **(A)** Adhesion abilities, **(B)** invasion abilities, **(C)** relative mRNA expression of IL-6 and IL-8 as analyzed by real-time RT-PCR, and **(D)** survival of bacteria in macrophage RAW264.7. The abilities to adhere to and invade A549 and NCI-H292 cells and intra-macrophage survival were determined as described in the Section “Materials and Methods.” The adhesion, invasion, and survival abilities of wild-type were set at 100%, and the other data are reported relative to these values. All data represent the average of three independent experiments with the SD. ^∗^*p* < 0.05; ^∗∗^*p* < 0.01; ^∗∗∗^*p* < 0.001.

*Acinetobacter baumannii* colonizes biotic surfaces by adherence and invasion to host cells to evade immune attacks and subsequent bacterial persistence (Lee et al., 2006). To test the role of Hfq in *A. baumannii* virulence, the ability of wild-type and Δhfq mutant cells to adhere to and invade A549 lung adenocarcinoma cells and NCI-H292 human bronchial epithelial cells was assessed. Δhfq exhibited impaired abilities to adhere to and invade A549 and NCI-H292 cells (**Figures 7A,B**). IL-8 and IL-6, which cause neutrophil infiltration and non-resolving inflammation, are key cytokines contributing to bacterial elimination by host cells and are expressed in the epithelial lining (Oglesby et al., 2015). IL-6 acts as both a pro-inflammatory cytokine and an anti-inflammatory myokine (Scheller et al., 2011), and IL-8 plays an important role in acute inflammation (Baggiolini and Clark-Lewis, 1992). Because Hfq plays a role in *A. baumannii* adherence and invasion of lung epithelial cells, we examined the production of IL-8 and IL-6 by A549 and NCI-H292 cells challenged with wild-type or Δhfq mutants. As shown in **Figure 7C**, IL-8 expression was significantly increased (*p* < 0.001) in both cells upon exposure to ΔHfq. However, IL-6 expression was significantly decreased (*p* < 0.01) in A549 cells but increased (*p* < 0.05) in NCI-H292 cells upon exposure to the Δhfq mutant compared with the WT and Δhfqc strains. This result implies that *hfq* deletion may stimulate IL-8 in A549 and NCI-H292 cells. Moreover, the deletion of *hfq* on IL-6 expression in both cells is different.

### Deletion of *hfq* Increased Killing of *A. baumannii* by Macrophage Cells

In order to understand if Hfq is involved in the innate ability of macrophage to eliminate *A. baumannii*, we co-cultured macrophages with bacteria, killed external bacteria with streptomycin, and assessed the survival of internalized *A. baumannii* after streptomycin treatment by lysing macrophage cells. As shown in **Figure 7D**, the relative survival of *hfq* mutant was reduced to 0.1% at 2 h after streptomycin treatment. The Hfq-complemented strain was partially restored the survival pattern compared to the wild-type.

## Discussion

Wide adaptation in the hospital environment enables microorganisms such as *A. baumannii* to become public healthcare-associated issues (Tang et al., 2015). Many studies have demonstrated that Hfq is associated with the timely adaptation to the environment and virulence of several pathogens (Sonnleitner et al., 2003; Chao and Vogel, 2010; Wang et al., 2014). *hfq* mutants have variable phenotypes among different bacteria. For example, improvement of motility was noted in a *Yersinia hfq* mutant (Schiano et al., 2010), but *Salmonella*, *P. aeruginosa*, and *E. coli* were impaired in motility upon loss of the *hfq* gene (Sonnleitner et al., 2003; Sittka et al., 2007; Kulesus et al., 2008). In addition, loss of the *hfq* gene decreases RpoS production in *Salmonella* and *E. coli* but not *V. cholerae* (Ding et al., 2004). These results show that the function of Hfq may be unique in each bacterial species. A previous study concerning about the hfq effect on *A. baylyi* showed that deletion of the complete *hfq* open reading frame resulted in severe reduction of growth (Schilling and Gerischer, 2009), which is consistent with our result. Herein, we first described the characteristics of *A. baumannii* Hfq involved in environmental adaptation and virulence. The *hfq* mutation led to pleiotropic phenotypic effects, including changes in stress tolerance, biofilm formation, cell morphology, epithelial cell adhesion and invasion, and survival in macrophage. The partial complementation in *bauA*, *groeL*, and *ompA* expression and cell adhesion, invasion, and intra-macrophage survival ability may reflect a gene dosage effect. All the pleiotropic effect could be explained by indirect consequences resulting from the loss of *hfq*.

Alterations of biofilm formation and colony morphology due to deletion of *hfq* have been demonstrated in several bacteria (Deng et al., 2016). However, reports of the precise visualization of cell morphology such as OMVs and fimbriae upon deletion of *hfq* remain limited. OMVs are an important vehicle designed to deliver effector molecules such as OmpA to host cells that contribute to pathogenesis during *A. baumannii* infection (Kwon et al., 2009; Jin et al., 2011). Our study reveals that the total proteins of OMVs are greatly reduced upon loss of the *hfq* gene. In *E. coli*, OmpA is downregulated by hfq-binding sRNA (Guillier et al., 2006). Based on our result (**Figure 2C**), we suggest that Hfq is necessary for OmpA expression.

Bacterial fimbriae have been recognized as mediators of initial host–pathogen interactions important for the progression of Gram-negative bacterial diseases (Kline et al., 2010). In addition, fimbriae have been implicated in other functions, such as phage-binding, DNA transfer, biofilm formation, cell aggregation, host cell invasion, and twitching motility (Proft and Baker, 2009). Several types of fimbriae have been described in *A. baumannii* (Tomaras et al., 2003; Harding et al., 2013). Type I fimbriae encoded by the CsuA/BABCDE chaperone-usher fimbria assembly system are involved in biofilm formation but are less important for adherence to mammalian cells (de Breij et al., 2009). Type IV fimbriae are involved in natural transformation and twitching motility but not surface-associated motility (Harding et al., 2013). In addition to the CsuA/BABCDE chaperone-usher fimbria assembly system, A1S_1507 and A1S_2091 have been found in the pellicle matrix formed by *A. baumannii* at the air-liquid surface (Nait Chabane et al., 2014). Moreover, A1S_1507 is involved in biofilm formation (Rumbo-Feal et al., 2013). Our AFM images revealed that fimbriae were greatly reduced in the Δhfq mutant, implying that *A. baumannii* Hfq is required for fimbriae production. Moreover, reduction of fimbriae production in the Δhfq mutant may reduce biofilm formation and cell adhesion and invasion (**Figures 6**, **7**). Interestingly, we also found that Hfq modulates fimbriae formation in a different manner. We hypothesized that Hfq can simultaneously upregulate and downregulate functionally different fimbriae. In addition, we suggested that the reduction of biofilm formation in Δhfq mutants may be mainly due to the reduction of Csu fimbriae rather than A1S_1507.

The ability of *A. baumannii* to sense and react to environmental and host stress signals allows it to persist and disseminate in medical settings and human hosts. Stressors include iron, an essential micronutrient for almost all living cells and organisms that causes severe damage when not properly controlled, and chemical antiseptics normally used in human medicine (Fiester and Actis, 2013). Acinetobactin, a siderophore from *A. baumannii*, is critical for acquisition of iron from culture media and from host cells and tissues (Mihara et al., 2004). The acinetobactin gene cluster contains 18 genes divided among 7 operons and includes 10 *bas* (acinetobactin biosynthesis) genes, 6 *bau* (acinetobactin utilization) genes and 2 *bar* (acinetobactin release) genes. Our finding shows that Hfq might be involved in the downregulation of acinetobactin via destabilization of the *bauA* and *basD* transcripts. Our results also indicate that Hfq downregulates several stress-related outer membrane proteins (OMPs). NlpE, an outer membrane lipoprotein that activates the Cpx signaling pathway, plays a role in sensing and inducing responses to different stressors (Chao and Vogel, 2016). A1S_0820, a protein containing the LysM domain, participates in the remodeling of the cell wall as cells grow into stationary phase (Soares et al., 2010). CarO is an OMP implicated in antibiotic resistance and ornithine transport (Mussi et al., 2007). The most abundant OMPs, such as OmpC, OmpF, and OmpA, are downregulated by Hfq-binding sRNA in *E. coli* (Guillier et al., 2006), indicating that sRNA regulation may be important for balancing porin levels or responding to the environment. In addition to OMPs, other stress-related proteins, GroEL and UspA, were also downregulated by Hfq. The association of Hfq and those stress-related proteins remains to be determined. However, our results show that deletion of *hfq* results in the over-expression of stress-related molecules and hypersensitization to environmental stresses.

Changes in OMPs caused by *hfq* mutation have been demonstrated to induce cytokine production (Wang et al., 2014), which is important to eliminate bacterial pathogens by triggering inflammatory responses. *A. baumannii* OMVs may elicit a potent innate immune response via membrane proteins (Jun et al., 2013; Kaparakis-Liaskos and Ferrero, 2015). Although OMVs were greatly reduced in the Δhfq mutant, the over-expression of surface proteins is likely to alter the immunogenicity of this bacterium or virulence factors that stimulates immune response to promote bacterial clearance by the host.

## Conclusion

This study implicates Hfq as a pivotal coordinator of diverse regulatory circuits, including cell-surface and/or cellular components. We provide evidence that, similar to its roles in other bacteria, Hfq has pleiotropic effects on stress tolerance, biofilm formation, cell morphology, epithelial cell adhesion, and invasion in *A. baumannii*. Our study provides a promising start for the characterization of the detailed regulatory mechanisms of Hfq and Hfq-regulated sRNA in the virulence of *A. baumannii.*

## Author Contributions

H-YK, LH, and M-LL designed the research project. P-CL, H-HC, K-CC, and C-HT carried out the experiment. M-LL, H-YK, and C-HC wrote the manuscript. All authors have read and approved the final manuscript.

## Conflict of Interest Statement

The authors declare that the research was conducted in the absence of any commercial or financial relationships that could be construed as a potential conflict of interest.
