# Supplementary material for: Functional Characterization of Acinetobacter baumannii Lacking the RNA Chaperone Hfq
Source: Front Microbiol. 2017 Oct 27;8:2068. doi: 10.3389/fmicb.2017.02068 (PMC5663733; doi:10.3389/fmicb.2017.02068)
Supplement: Supplementary file 1 [file Table_1.DOCX]

| Table S1. Oligonucleotides used in this study | | |  |  |
| --- | --- | --- | --- | --- |
| Oligonucleotide name | Oligonucleotide sequence(5´to3´) | Reference or source | |  |
| Mutant construction | | |  |  |
| Up-*hfq*_F | CGATTGTTTTCGTCGTCTTGACCATCTTCA | This study | |  |
| *hfq*_*kan*_rpF | GGACGTGGGTTACGAGCTTAGAAAAACTCATCGAGCATC | This study | |  |
| *hfq*_*kan*_rpR | GATGCTCGATGAGTTTTTCTAAGCTCGTAACCCACGTCC | This study | |  |
| *kan*_*hfq*_rpF | GTTTCCCGTTGAATATGGCTCATAGTTTGACCTTTAGACATTTTTAACTC | This study | |  |
| *kan*_*hfq*_rpR | GAGTTAAAAATGTCTAAAGGTCAAACTATGAGCCATATTCAACGGGAAAC | This study | |  |
| ScF | CAGAAACCAGAACACTGCACAATGCTG | This study | |  |
| ScR | CGTTCTGTTGGTTATCGACAAGCTCTAG | This study | |  |
| Complementation cloning | | |  |  |
| hfq_Expr_XhoI_F | TTAACTCGAGATAAGCAAAATTTGCACACTGTCTTTTTA | This study | | |
| hfq_Expr_XbaI_R | TAGCTCTAGATTAATTAACGATTGTTTTCGTCGTCTTG | This study | | |
| Real-time RT-PCR |  |  | | |
| *hfq*_qF | GCGTGTTTGTAAACCATTTGACTTAC | This study | | |
| *hfq*_qR | CATCCCAGTTTCTATTTTCCTTGTTAAC | This study | | |
| CsuA/B_qF | AAAACTTCCGGTACTTGGAACAACGTAT | [1] | | |
| CsuA/B_qR | AAAATCAACAGGATCTGTTCCGTCACA | [1] | | |
| A1S_1507_qF | ACACCAACTCCGTTTGCTTT | This study | | |
| A1S_1507_qR | CTGACACTTCAAATAGCCAGGTT | This study | | |
| 16s rRNA_qF | CAGCTCGTGTCGTGAGATGT | [1] | | |
| 16s rRNA_qR | CGTAAGGGCCATGATGACTT | [1] | | |
| IL-6_qF | TGAGAGTAGTGAGGAACAAGCCAG | [2] | | |
| IL-6_qR | TGGCATTTGTGGTTGGGTC | [2] | | |
| IL-8_qF | ATAAAGACATACTCCAAACCTTTCCAC | [3] | | |
| IL-8_qR | AAGCTTTACAATAATTTCTGTGTTGGC | [3] | | |
| GAPDH_qF | CCATGTTCGTCATGGGTGTG | [2] | | |
| GAPDH_qF | GTGCAGGAGGCATTGCTGAT | [2] | | |
| BasD_qF | AGACCGCTTATGGTGGAAAGAA | This study | | |
| BasD_qR | CGATAGCCAAGTACTTAAACGATCAC | This study | | |
| BauA_qF | CTCGGATCATACCTCCCGTATC | This study | | |
| BauA_qR | TTTACCCAAAGCGCCAAATC | This study | | |
| UspA_qF | AAGCAATCAAAATTGGGAAAGC | This study | | |
| UspA_qR | TGTTCTAGTACGCTCAATCAATTCATT | This study | | |
| NlpE_qF | TTGCATCACCTTTACCAAGATAGG | This study | | |
| NlpE_qR | CACATACAGCCGAGAATTCTTTAGAC | This study | | |
| A1S_0820_qF | GGTAATGTAGATCATGTGGCACAAG | This study | | |
| A1S_0820_qR | TGATTGGCATCACCATAAAATTCTT | This study | | |
| CarO_F | CCTGCAGCTACATATAAGCCTTGAG | This study | | |
| CarO_R | TGGCGAGATGACTTATCAATTAATG | This study | | |
| OmpA_qF | CGAATACACGACGGTTCATAGC | This study | | |
| OmpA_qR | CGAATACACGACGGTTCATAGC | This study | | |
| GroEL_qF | AATCGCACGGCGTAAAATG | This study | | |
| GroEL_qR | AATCGCACGGCGTAAAATG | This study | | |

1. Chang KC, Kuo HY, Tang CY, Chang CW, Lu CW, Liu CC, Lin HR, Chen KH, Liou ML: **Transcriptome profiling in imipenem-selected Acinetobacter baumannii**. *BMC Genomics* 2014, **15**(1):815.

2. Fruchon S, Poupot M, Martinet L, Turrin CO, Majoral JP, Fournie JJ, Caminade AM, Poupot R: **Anti-inflammatory and immunosuppressive activation of human monocytes by a bioactive dendrimer**. *J Leukoc Biol* 2009, **85**(3):553-562.

3. Nhu QM, Shirey K, Teijaro JR, Farber DL, Netzel-Arnett S, Antalis TM, Fasano A, Vogel SN: **Novel signaling interactions between proteinase-activated receptor 2 and Toll-like receptors in vitro and in vivo**. *Mucosal Immunol* 2009, **3**(1):29-39.
