# Supplementary figures and images for: Functional Characterization of Acinetobacter baumannii Lacking the RNA Chaperone Hfq
Source: Front Microbiol. 2017 Oct 27;8:2068. doi: 10.3389/fmicb.2017.02068 (PMC5663733; doi:10.3389/fmicb.2017.02068)

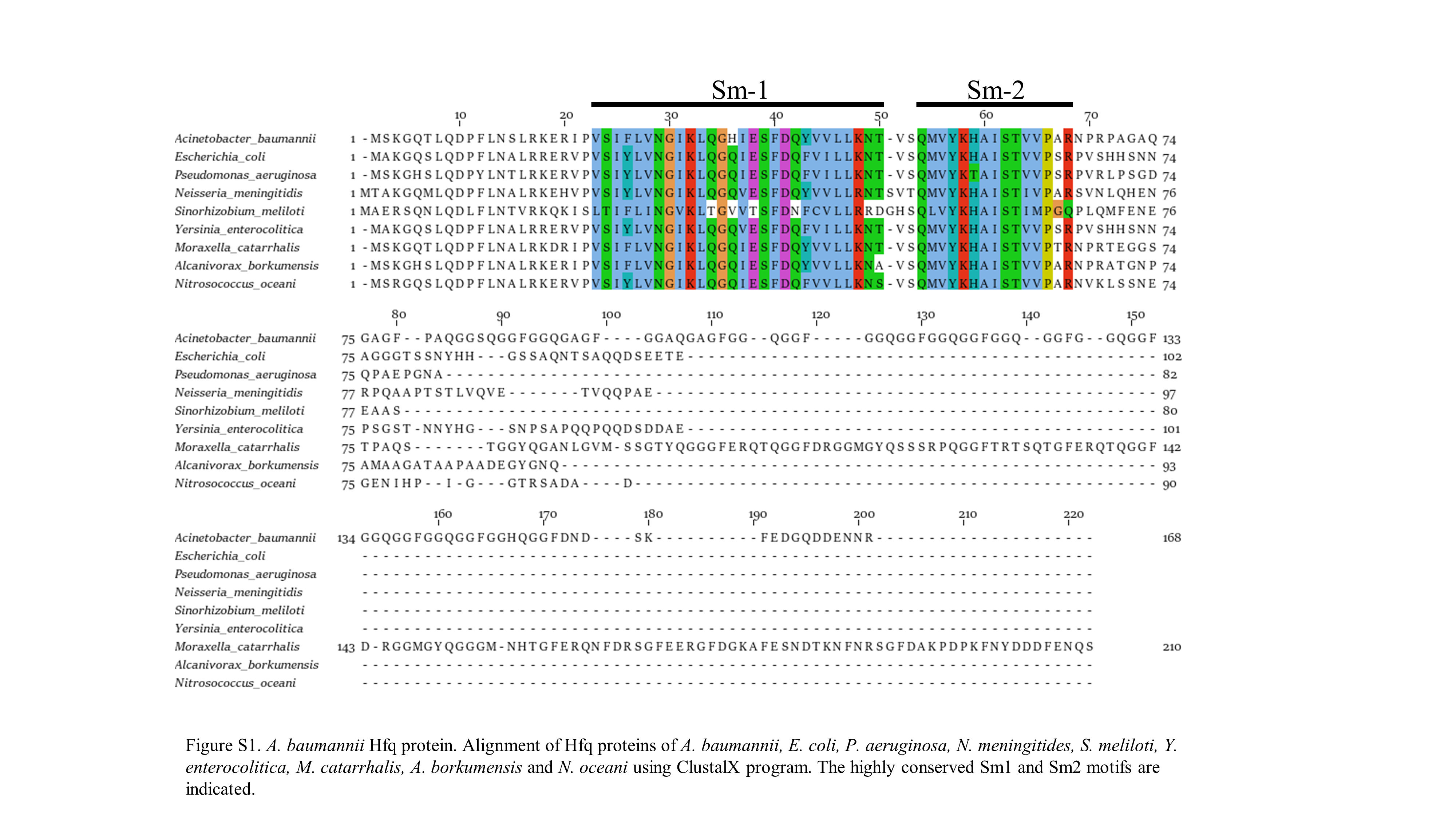

Supplement: Supplementary file 2 [file Image_1.TIF]
